# Brassinolide Alleviates Maize Silk Growth Under Water Deficit by Reprogramming Sugar Metabolism and Enhancing Antioxidant Defense

**DOI:** 10.3390/plants15010139

**Published:** 2026-01-03

**Authors:** Jinrong Xu, Zhicheng Cheng, Li Dai, Wangjing Li, Liyuan Chen, Gatera Anicet, Yi Yu, Youhong Song

**Affiliations:** 1 School of Agronomy, Anhui Agricultural University, Hefei 230036, China; xjrong@ahau.edu.cn (J.X.); dailiyouxiang2023@163.com (L.D.); lwj@stu.ahau.edu.cn (W.L.); chenliyuan@caas.cn (L.C.); gateran7@stu.ahau.edu.cn (G.A.); yyyiyu@stu.ahau.edu.cn (Y.Y.); 2 Ministry of Education Key Laboratory of Crop Physiology, Ecology and Genetic Breeding, Jiangxi Agricultural University, Nanchang 330045, China; czc1123@stu.jxau.edu.cn; 3 Centre for Crop Science, Queensland Alliance for Agriculture and Food Innovation, The University of Queensland, Brisbane, QLD 4350, Australia

**Keywords:** *Zea mays* L., reproductive-stage water deficit, phytohormone, silk elongation, sucrose metabolism, transcriptome

## Abstract

Reproductive-stage drought arrests silk elongation, causing a greater anthesis-silking interval and subsequent kernel loss in maize. Exogenous brassinolide (BR) is known to increase drought tolerance; however, its influence on silk growth under water deficit remains unresolved. Here, we subjected maize to drought before tassel emergence (V13) and then applied foliar BR at concentrations of 0, 0.1, 0.5, or 1 mg mL^−1^, with distilled water-sprayed plants serving as controls. Silk elongation under water-deficit stress was partially restored by 0.1 and 0.5 mg mL^−1^ BR but suppressed by 1 mg mL^−1^, with 0.5 mg mL^−1^ increasing silk length by 2.9-fold compared to the stress control, recovering it to 26.5% of the well-watered level. This protection was underpinned by elevated antioxidant capacity (POD, SOD, and CAT by 31–77%, 12–46%, and 20–33%, respectively) and a 25–76% rise in proline relative to the distilled water-sprayed, which collectively curtailed oxidative damage, as evidenced by 36–67% reductions in O_2_^−^ and H_2_O_2_ levels and a 24% decrease in MDA content. Critically, BR reprogrammed sugar metabolism: sucrose phosphate synthase (SPS) activity declined, while sucrose synthase (SS-I) and vacuolar invertase (VIN) activities surged, thereby shifting carbon partitioning from sucrose toward hexoses to sustain energy supply for silk growth. Genome-wide RNA-seq identified 6171 upregulated and 3295 downregulated genes, significantly enriched in 20 pathways, including starch/sucrose metabolism, glycolysis/gluconeogenesis, and phenylpropanoid biosynthesis. The expression of key genes, including sucrose invertase (INV) and hexokinase (HK), was significantly upregulated by 2.4- to 8.7-fold and 2.3- to 4.0-fold, respectively, compared to the distilled water-sprayed control. This multi-level analysis demonstrates that BR mitigates drought-induced silk growth arrest by orchestrating antioxidant defense, osmotic regulation, and metabolic reprogramming into a coordinated network, providing mechanistic insights into brassinosteroid-mediated reproductive stress adaptation in maize.

## 1. Introduction

Maize (*Zea mays* L.), as an important crop for food, feed, and industrial materials, is sensitive to drought stress during growth and development [1,2,3,4,5]. Under climate change, drought stress is increasingly threatening global maize grain yields [6,7]. For example, in the Huang-Huai-Hai region of China, a key area for summer maize cultivation, maize is highly susceptible to drought stress during the flowering period. During the flowering stage, drought stress severely inhibits silk elongation, while anthesis remains largely unaffected [8]. This physiological asynchrony causes an extended anthesis-silking interval (ASI). As silks are crucial for capturing pollen, the prolonged ASI leads to incomplete pollination and fertilization failure, ultimately causing grain yield reductions [9,10,11]. Thus, developing effective strategies for mitigating drought-induced losses is urgent.

The application of exogenous plant growth regulators (PGRs) has become a promising strategy in enhancing crop drought tolerance [12,13,14]. For instance, abscisic acid (ABA) induces rapid stomatal closure to reduce water loss, while salicylic acid (SA) primes systemic defense responses [15,16,17,18]. Other compounds, such as jasmonates, polyamines, and glycine betaine, also improve stress resilience through diverse mechanisms [19,20,21,22]. Among them, brassinolide (BR) has received widespread attention due to its broad-spectrum effects in regulating growth, development, and stress adaptation [23,24]. For example, foliar spraying 0.1 mg/L BR enhanced maize drought tolerance by improving photosynthetic performance, increasing the activities of antioxidant enzymes (SOD, POD, and CAT), and elevating the proline content [25]. The BR application enhanced rice drought tolerance by boosting osmoprotectant levels (phenolics, anthocyanins, and proline) to maintain cellular osmotic balance effectively [26]. Additionally, BR spraying promoted rice spikelet development under drought stress through activation of cell-cycle-related gene expression (e.g., *CYCD3;1*, *CYCB1;1*), promoting cell division [27]. BR spraying improved latex yield in drought-stressed rubber trees by increasing net photosynthetic rate and promoting the accumulation of glucose, starch, and soluble sugars, thus enhancing energy reserves [28]. Furthermore, BR spraying also enhanced wheat drought resistance by augmenting antioxidant enzyme activities in reducing the accumulation of reactive oxygen species (ROS), specifically superoxide anions (O_2_^−^) and hydrogen peroxide (H_2_O_2_), thereby minimizing oxidative cellular damage [29]. Collectively, BR plays critical roles in enhancing plant drought tolerance through mechanisms involving osmotic adjustment, antioxidant defense, energy metabolism, and regulation of growth processes.

However, little is known about whether BR application during the early reproductive stage can alleviate the inhibition of water-deficit stress on silk elongation in the female ear, as well as its underlying mechanism of regulating physiological metabolism. During reproductive growth, silk elongation critically depends on a sustained carbon and energy supply, with sucrose serving as the primary transported carbohydrate [30,31]. It can be subsequently hydrolyzed into glucose and fructose through cell wall invertase (CWIN), vacuolar invertase (VIN), and sucrose synthase (SUS), providing both carbon skeletons and ATP-generating substrates required for cell expansion [32,33,34]. However, drought stress disrupts this process by impairing sucrose phloem unloading into silks and suppressing the CWIN, VIN, and SUS activities [35]. This disruption leads to an imbalance characterized by sucrose accumulation and hexose depletion within the silk, creating a carbon and energy deficit that ultimately restricts silk elongation. We therefore hypothesize that the key mechanism by which BR alleviates water-deficit-induced silk arrest may not solely rely on its general antioxidant or osmotic functions, but rather on its ability to specifically reprogram silk sucrose metabolism, thereby restoring the hexose-driven energy supply and turgor pressure essential for elongation.

Within the content, given these BR-mediated processes, we hypothesize that exogenous BR may alleviate water-deficit-induced silk elongation inhibition through a multifaceted physiological mechanism that not only enhances antioxidant defense and promotes osmotic regulation to maintain cellular homeostasis, but more importantly, reprograms sucrose metabolism to secure sufficient carbon and energy supply for cell expansion. Thus, this study aims to (i) quantify the mitigating effects of exogenous BR on silk elongation under reproductive-stage water-deficit stress and (ii) determine the optimal BR concentration in relieving the water-deficit stress impact and evaluate the associated biochemical properties.

## 2. Materials and Methods

### 2.1. Experimental Location and Design

A pot trial was conducted in 2024 at the Nongcui Garden of Anhui Agricultural University, China. Maize hybrid Zhengdan958 (ZD958) was used in the study. The loam soil was used for the test soil (organic matter, 13.53 g∙kg^−1^; total nitrogen, 1.04 g∙kg^−1^; fast-acting nitrogen, 78.22 mg∙kg^−1^; fast-acting phosphorus, 23.86 mg∙kg^−1^; fast-acting potassium, 66.87 mg∙kg^−1^). Each pot contains 20 kg of dry soil, 3.54 g of urea, 1.36 g of diammonium phosphate, and 6.08 g of potassium sulfate. Fertilization management adopts a combination of base fertilizer and topdressing: 2.88 g of compound fertilizer (N-P_2_O_5_-K_2_O = 14%–16%–15%) was applied to each pot as a base dose, followed by 3.52 g of urea (nitrogen content of 46%) applied as top dressing at the V10 stage. The mixed soil was placed in 48 cm × 30 cm pots with six drainage holes. Then, five seeds were sown in each pot. The experiment followed a completely randomized design with 3 replicates per treatment. All treatments included WW (well-watered control), DS (water-deficit stressed without foliar spray), DS+H_2_O (water-deficit stressed with distilled water spray serving as spray control), DS+BR1 (water-deficit stressed with 0.1 mg mL^−1^ BR), DS+BR2 (water-deficit stressed with 0.5 mg mL^−1^ BR), and DS+BR3 (water-deficit stressed with 1 mg mL^−1^ BR).

At the maize V11 stage, water control operations were initiated using drying and weighing methods, gradually reducing soil moisture by stopping the water supply. When the maize reached the V13 stage, soil moisture content was maintained at 55% ± 5% of soil field capacity to establish moderate water-deficit stress. Concurrently, foliar spraying treatments were applied: (i) distilled water (DS+H_2_O, sprayed as the control); (ii) BR (0.1, 0.5, 1 mg mL^−1^). Distilled water spraying (DS+H_2_O) served as a control to specifically evaluate the water deficit-relief effect mediated by BR application. Exogenous BR and distilled water were applied on the 1st and 3rd days of water deficit imposition, with each pot receiving 250 mL. The 10th, 20th, and 30th ovule positions, numbered acropetally, were defined as the basal, middle, and top silks, respectively. Then, to specifically assess the response of the most water deficit-sensitive reproductive tissue, top silks were collected on the tenth day of water-deficit stress, immediately frozen in liquid nitrogen, and stored at −80 °C.

### 2.2. Measurement of Silk Length

Silk length was measured using a high-precision vernier caliper. These values were measured in centimeters.

### 2.3. Assay of Peroxide Content and Antioxidant Enzyme Activity in Silks

Hydrogen peroxide (H_2_O_2_) and superoxide anion (O_2_-) were quantified using commercial kits (G0168W for H_2_O_2_ and G0116W48 for O_2_^−^, Suzhou Grace Biotechnology Co., Ltd., Suzhou, China). For H_2_O_2_, 0.1g of fresh silk was homogenized in 1 mL of pre-chilled acetone and centrifuged. Subsequently, 64 µL of the supernatant was mixed sequentially with the assay reagents. After incubation at room temperature for 5 min, the absorbance was measured at 510 nm. The O_2_^−^ content was determined following an analogous procedure specified in its kit, with absorbance measured at 530 nm.

The activities of peroxidase (POD), superoxide dismutase (SOD), and catalase (CAT) were assayed using their respective commercial kits (G0107W for POD, G0101W for SOD, and G0105W for CAT, Suzhou Grace Biotechnology Co., Ltd, Suzhou, China). For each assay, 0.1 g of fresh silk was homogenized in 1 mL of the specified extraction buffer and centrifuged to obtain the supernatant. POD activity was determined by monitoring the increase in absorbance at 470 nm. SOD activity was measured using the WST-8 method. The inhibition of the formazan dye formation was monitored at 450 nm. CAT activity was assayed by measuring the decomposition at 240 nm.

### 2.4. Determination of MDA Content in Silks

Malondialdehyde (MDA) was determined according to the thiobarbituric acid (TBA) method [36]. An amount of 0.1 g of fresh silk was homogenized in 3 mL of 5% trichloroacetic acid (TCA) and centrifuged. Then the supernatant was collected, mixed with 2 mL of 0.67% TBA, and incubated at 95 °C for 30 min. After cooling, the mixture was centrifuged, and the absorbance of the resulting supernatant was measured at 532 and 600 nm.

### 2.5. Determination of Proline Contents in Silks

The proline content (Pro) was quantified according to the sulfosalicylic acid method [37]. An amount of 0.1 g of silk was mixed with 3 mL of 3% sulfosalicylic acid solution and centrifuged. A 1 mL aliquot of the supernatant was mixed with 1 mL of glacial acetic acid and 1 mL of acidified ninhydrin reagent. The mixture was heated at 100 °C for 40 min in a water bath. After cooling to room temperature, the reaction mixture was extracted with 2 mL of toluene. Following phase separation, the absorbance of the upper organic phase was measured at 520 nm using a spectrophotometer.

### 2.6. Determination of Sugar Contents in Silks

The total soluble sugar, sucrose, fructose, and glucose contents were determined according to the methods of Yu et al. [38]. 0.1 g of silk was extracted with 80% alcohol and incubated in a water bath at 80 °C for 30 min. The extract was treated with activated carbon for decolorization and centrifuged to obtain the clear supernatant for subsequent assays. Total soluble sugar content was determined using the anthrone-sulfuric acid method. An aliquot of the extract was mixed with anthrone reagent and concentrated sulfuric acid. After boiling, the resulting green-colored complex was measured at 620 nm. Sucrose was quantified by the resorcinol method. The supernatant was reacted with resorcinol in concentrated hydrochloric acid (HCl). After heating, the developed cherry-red color was measured at 480 nm. Fructose was assayed using a resorcinol-thiourea method specific for ketoses. The extract was mixed with a resorcinol-thiourea reagent in hydrochloric acid. After heating, the absorbance of the yellow-orange product was measured at 480 nm. Glucose content was determined enzymatically using a commercial Glucose Assay Kit (Beijing Solarbio Science & Technology Co., Ltd., Beijing, China). The assay follows the glucose oxidase-peroxidase (GOD-POD) principle, where glucose is oxidized to produce hydrogen peroxide, which reacts with a chromogen to form a red quinoneimine dye. The absorbance was measured at 505 nm.

### 2.7. Determination of Sugar Metabolism Level in Silks

The activities of cell wall invertase (CWIN), vacuolar invertase (VIN), sucrose phosphate synthase (SPS), and sucrose synthase (SS-I, catabolic direction) were assayed using commercial enzyme assay kits (Suzhou Grace Biotechnology Co., Ltd., Suzhou, China; Cat. Nos. G0518W, G0517W, G0515W, and G0513W, respectively) following the manufacturer’s instructions. Before enzyme extraction, silk samples were pre-washed with pre-chilled 95% alcohol to remove endogenous soluble sugars, thereby mitigating background interference. WIN and VIN activities were determined by quantifying the reducing sugars released from sucrose hydrolysis. The enzymes were differentiated based on extraction: CWIN was extracted from the insoluble pellet, while VIN was obtained from the supernatant. The reaction mixtures were incubated at 37 °C for 20 min, and the resulting reducing sugars were measured at 540 nm using the 3,5-dinitrosalicylic acid (DNS) method. Activities were calculated based on a glucose standard curve. SPS activity was assayed by monitoring the synthesis of sucrose phosphate from fructose-6-phosphate and UDP-glucose. The reaction was performed at 37 °C for 20 min, and the product was determined colorimetrically at 480 nm using the resorcinol method. SS-I activity (catabolic direction) was determined by measuring the release of fructose from the cleavage of sucrose in the presence of UDP. The reaction mixture was incubated at 37 °C for 30 min, and the reducing sugar content was measured at 540 nm using the DNS method. SS-I activity was calculated using a fructose standard curve. One unit (U) of enzyme activity was defined as the amount of enzyme producing 1 µg of product per minute per g of fresh weight, where the reaction rate is proportional to the enzyme concentration.

### 2.8. RNA-Seq Analyses

The silks were collected on the tenth day of water deficit for transcriptome analysis. Total RNA was extracted using the Trizol reagent kit (Invitrogen, Carlsbad, CA, USA) and treated with RNase-free DNase I (Takara Bio Inc., Kyoto, Japan) to remove genomic DNA. RNA concentration and purity were measured with a NanoDrop ND-1000 spectrophotometer (NanoDrop, Wilmington, DE, USA), and integrity was evaluated on an Agilent 2100 Bioanalyzer (Agilent, Santa Clara, CA, USA) and confirmed by agarose-gel electrophoresis. All samples met the following quality thresholds: concentration > 50 ng/μL, RIN > 7.0, OD260/280 > 1.8, and total RNA > 1 μg. Sequencing libraries were prepared following the strand-specific protocol of the NEBNext UltraT™ RNA Library Prep Kit for Illumina New England Biolabs, Ipswich, MA, USA). Poly(A)+ mRNA was enriched using Dynabeads Oligo(dT) (Thermo Fisher, Waltham, MA, USA) and fragmented with the NEBNext Magnesium RNA Fragmentation Module. First-strand cDNA was synthesized from the fragmented RNA using SuperScript II Reverse Transcriptase (Invitrogen, Carlsbad, CA, USA), followed by second-strand synthesis with E. coli DNA polymerase I and RNase H (New England Biolabs, Ipswich, MA, USA). The double-stranded cDNA was blunt-ended by incorporation of dUTP (ThermoFisher, Waltham, MA, USA), and A-tailing was performed to enable ligation of adapters with T-overhangs. The resulting libraries were size-selected (350 bp) using magnetic beads and amplified by PCR. Finally, 150 bp paired-end sequencing was carried out on an Illumina NovaSeq 6000 platform (LC-Bio Technology Co., Ltd., Hangzhou, China).

Then, Cutadapt v1.9 was used to remove adapter sequences and low-quality reads (Phred Q score < 20). Clean reads were then aligned to the *Zea mays* reference genome B73 (https://www.maizegdb.org/genome/assembly/Zm-B73-REFERENCE-NAM-5.0, accessed on 9 December 2024) using HISAT2 [39] (https://daehwankimlab.github.io/hisat2/, version is hisat2-2.0.4, accessed on 9 December 2024). Transcriptome assembly and quantification were conducted using String Tie [40] (https://ccb.jhu.edu/software/stringtie/, version stringtie-1.3.4d. Linux_x86_64, accessed on 9 December 2024), and the merged assembly was annotated with gffcompare (http://ccb.jhu.edu/software/stringtie/gffcompare.shtml, accessed on 9 December 2024). Gene expression levels were normalized and expressed as Fragments Per Kilobase of exon model per Million mapped reads (FPKM) using the ballgown package [41] (http://www.bioconductor.org/packages/release/bioc/html/ballgown.html, accessed on 9 December 2024). Differential expression analysis was performed with DESeq2 [42] (https://bioconductor.org/packages/release/bioc/html/DESeq2.html, accessed on 9 December 2024). And genes with fold change > 2 or fold change < 0.5, with an adjusted *p*-value < 0.05, were considered significantly differentially expressed for subsequent Gene Ontology (GO) and Kyoto Encyclopedia of Genes and Genomes (KEGG) enrichment analyses.

### 2.9. RT-qPCR Validation

To validate the RNA-seq data, fifteen differentially expressed genes were selected for qRT-PCR analysis. For cDNA synthesis, 1 μg of total RNA from each sample was reverse-transcribed using the HiScript ^®^ III RT Super Mix reverse transcription kit (Vazyme, Nanjing, China). qRT-PCR reactions were performed in 20 μL volumes containing SYBR Green Master Mix (Biosharp, Beijing, China) and gene-specific primer pairs on a CFX96 Real-Time PCR Detection System (Bio-Rad Laboratories, Inc., Hercules, CA, USA). The thermal cycling protocol was as follows: initial denaturation at 95 °C for 30 s, followed by 40 cycles of 95 °C for 10 s and 60 °C for 30 s. Actin serves as an internal control to normalize gene expression levels and calculate the relative expression levels of each DEG using the 2^(−ΔΔCt) method [43].

### 2.10. Statistical Analysis

Statistical analysis was conducted utilizing one-way analysis of variance (ANOVA) in SPSS 24.0. This was followed by Tukey’s test to determine significant differences (*p* ≤ 0.05). Graphical representations were created using GraphPad Prism 9.5.

## 3. Results

### 3.1. Effect of Exogenous BR on Silk Length Under Water-Deficit Stress

**Figure 1:**
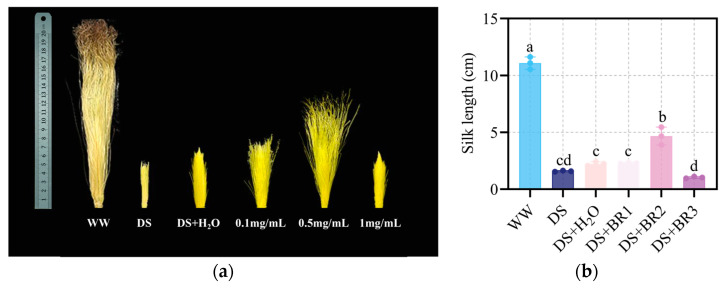
Effects of exogenous BR on the growth performance of maize silk. (**a**) Phenotype of maize silk after spraying BR under water-deficit stress; (**b**) Silk length, i.e., WW, DS, DS+H_2_O, DS+BR1, DS+BR2, and DS+BR3. WW, well-watered; DS, water-deficit stress; DS+H_2_O, as spraying control, spraying distilled water under water-deficit stress; DS+BR1, DS+BR2, and DS+BR3, water-deficit stress with foliar spray of 0.1, 0.5, and 1 mg mL^−1^ BR, respectively. The different letters marked on the bars indicate significant differences with *p* < 0.05, according to one-way ANOVA and Tukey’s test. Data are means ± SD (*n* = 3).

The changes in silk growth performance under different concentrations of BR application are shown in Figure 1. Water-deficit stress significantly decreased the silk length by 84.75% compared to WW. Under water-deficit stress, the silk length was increased by 41.96% with spraying distilled water compared with DS. Spraying 0.1 and 0.5 mg mL^−1^ BR further increased the silk length by 3.43% and 107.89% relative to DS+H_2_O, respectively. In contrast, spraying 1 mg mL^−1^ BR exacerbated water deficit-induced inhibition of silk elongation. Among them, the 0.5 mg mL^−1^ BR was most effective in alleviating water deficit-induced inhibition of silk elongation.

### 3.2. Effect of Exogenous BR on Oxidative Stress and Membrane Damage in Water-Deficient Silk

**Figure 2:**
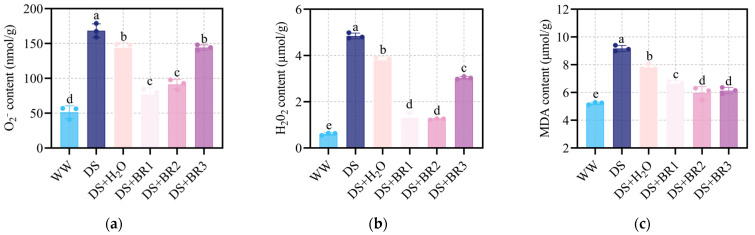
Content of (**a**) superoxide anion (O_2_^−^), (**b**) hydrogen peroxide (H_2_O_2_), and (**c**) malondialdehyde (MDA) in maize silk under water-deficit stress treated with different foliar spraying of BR, i.e., WW, DS, DS+H_2_O, DS+BR1, DS+BR2, and DS+BR3. WW, well-watered; DS, water-deficit stress; DS+H_2_O, as spraying control, spraying distilled water under water-deficit stress; DS+BR1, DS+BR2, and DS+BR3, water-deficit stress with foliar spray of 0.1, 0.5, and 1.0 mg mL^−1^ BR, respectively. The different letters marked on the bars indicate significant differences with *p* < 0.05, according to one-way ANOVA and Tukey’s test. Data are means ± SD (*n* = 3).

The changes in the O_2_^−^ and H_2_O_2_ and MDA in silk with different concentrations of BR application under water-deficit stress are shown in Figure 2. Water-deficit stress significantly increased the ROS accumulation and membrane damage. Spraying distilled water significantly reduced O_2_^−^, H_2_O_2_, and MDA content by 15%, 31%, and 15% compared with DS, respectively. Under water-deficit stress, all BR concentrations further suppressed ROS accumulation and membrane damage over the DS+H_2_O. 0.1, 0.5, and 1 mg mL^−1^ BR reduced O_2_^−^ content by 46.84%, 36.44%, and 11.11% (Figure 2a) and decreased H_2_O_2_ content by 65.3%, 66.9%, and 20.1%, respectively (Figure 2b). Meanwhile, spraying 0.1, 0.5, and 1 mg mL^−1^ BR reduced MDA content by 15%, 24%, and 21%, respectively (Figure 2c). Among them, the 0.5 mg mL^−1^ BR consistently exhibited the most potent activity in mitigating both the oxidative stress and its downstream damage to membranes.

### 3.3. Effect of Exogenous BR on Antioxidant Enzyme Activities in Water-Deficient Silk

**Figure 3:**
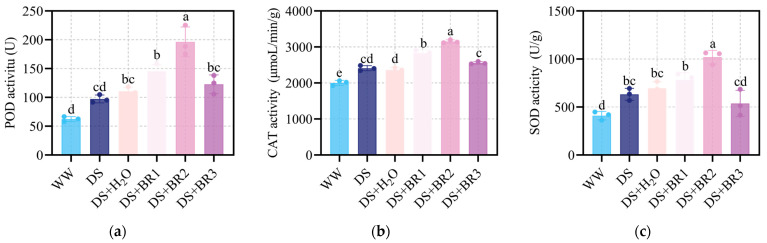
Activity of (**a**) peroxidase (POD), (**b**) superoxide dismutase (SOD), and (**c**) catalase (CAT) in maize silk under water-deficit stress treated with different foliar spraying of BR, i.e., WW, DS, DS+H_2_O, DS+BR1, DS+BR2, and DS+BR3. WW, well-watered; DS, water-deficit stress; DS+H_2_O, as spraying control, spraying distilled water under water-deficit stress; DS+BR1, DS+BR2, and DS+BR3, water-deficit stress with foliar spray of 0.1, 0.5, and 1.0 mg mL^−1^ BR, respectively. The different letters marked on the bars indicate significant differences with *p* < 0.05, according to one-way ANOVA and Tukey’s test. Data are means ± SD (*n* = 3).

The changes in activities of POD, CAT, and SOD in silk with different concentrations of BR application under water-deficit stress are shown in Figure 3. Water-deficit stress significantly enhanced the activities of POD, SOD, and CAT. Spraying distilled water did not significantly enhance antioxidant capacity relative to DS. However, compared to DS+H_2_O, spraying 0.1 and 0.5 mg mL^−1^ BR further significantly elevated the POD, SOD, and CAT activities, with 0.5 mg mL^−1^ BR being the most effective. Among them, spraying 0.1 mg mL^−1^ BR increased the activities of POD, SOD, and CAT by 31.36%, 12.41%, and 20.05%, respectively. Spraying 0.5 mg mL^−1^ BR increased POD by 77.20%, SOD by 46.49%, and CAT by 33.43%. In contrast, compared to DS+H_2_O, spraying 1 mg mL^−1^ BR not only had no significant difference in POD activity (Figure 3a) but also suppressed SOD activity and only slightly increased CAT activity by 8.27% (Figure 3b,c).

### 3.4. Effect of Exogenous BR on Proline Content in Water-Deficit-Stressed Silk

**Figure 4:**
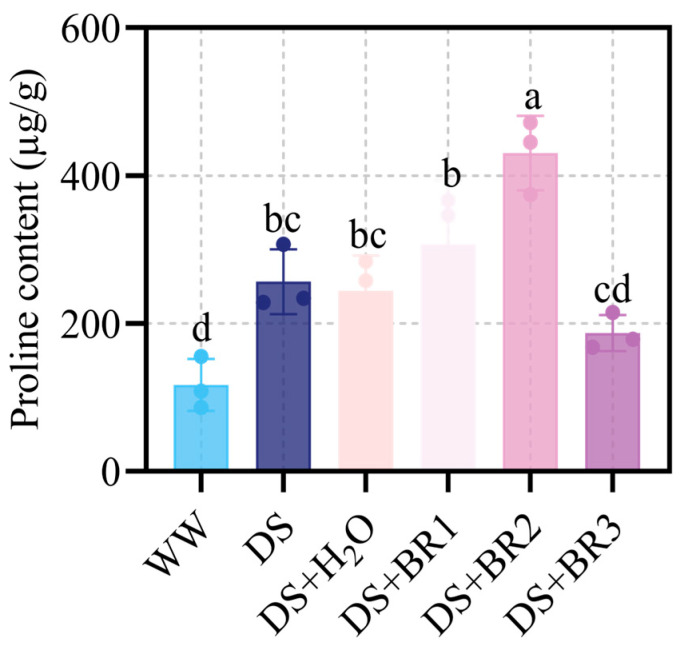
Proline content of maize silk under water-deficit stress treated with different foliar spraying of BR, i.e., WW, DS, DS+H_2_O, DS+BR1, DS+BR2, and DS+BR3. WW, well-watered; DS, water-deficit stress; DS+H_2_O, as spraying control, spraying distilled water under water-deficit stress; DS+BR1, DS+BR2, and DS+BR3, water-deficit stress with foliar spray of 0.1, 0.5, and 1.0 mg mL^−1^ BR, respectively. The different letters marked on the bars indicate significant differences with *p* < 0.05, according to one-way ANOVA and Tukey’s test. Data are means ± SD (*n* = 3).

The changes in proline content in silk with different concentrations of BR application under water-deficit stress are shown in Figure 4. Water-deficit stress significantly increased proline content by 91.2% related to WW. Compared with DS, spraying distilled water did not significantly increase proline content. However, spraying 0.1 and 0.5 mg mL^−1^ BR increased proline content by 25.68% and 76.01% relative to DS+H_2_O, respectively. The 1 mg mL^−1^ BR significantly reduced proline content by 22.42%. Among them, the highest proline content was observed under 0.5 mg mL^−1^ BR.

### 3.5. Effect of Exogenous BR on Sugar Contents and Sugar Metabolism in Water-Deficit-Stressed Silk

**Figure 5:**
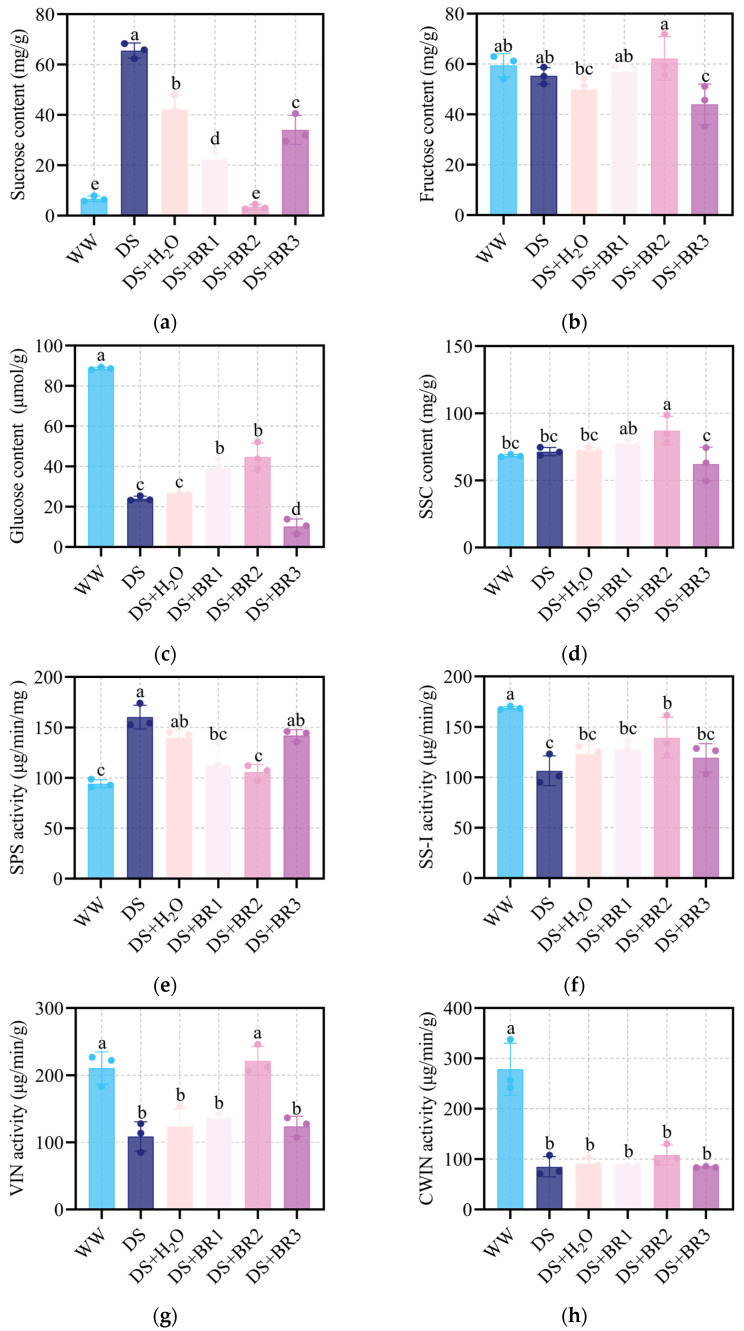
Content of (**a**) sucrose, (**b**) fructose, (**c**) glucose, (**d**) soluble sugar content, (**e**) sucrose phosphate synthase activity (SPS), (**f**) sucrose synthase activity (catabolic direction; SS-I), (**g**) soluble acidic vesicle invertase activity (VIN), and (**h**) cell wall acidic invertase activity (CWIN) in maize silk under water deficit-stress treated with different foliar spraying of BR, i.e., WW, DS, DS+H_2_O, DS+BR1, DS+BR2, and DS+BR3. WW, well-watered; DS, water-deficit stress; DS+H_2_O, as spraying control, spraying distilled water under water-deficit stress; DS+BR1, DS+BR2, and DS+BR3, water-deficit stress with foliar spray of 0.1, 0.5, and 1.0 mg mL^−1^ BR, respectively. The different letters marked on the bars indicate significant differences with *p* < 0.05, according to one-way ANOVA and Tukey’s test. Data are means ± SD (*n* = 3).

The changes in sugar content and sugar metabolism enzymes in silk with different concentrations of BR application under water-deficit stress are shown in Figure 5. Water-deficit stress significantly increased sucrose content and reduced hexose accumulation. Sucrose, fructose, glucose, and soluble sugar contents showed minimal effect in spraying distilled water relative to the DS. Compared to DS+H_2_O, 0.1, 0.5, and 1 mg mL^−1^ BR decreased sucrose by 46.65%, 91.91%, and 19.13%, respectively (Figure 5a). Meanwhile, under foliar spraying of 0.1, 0.5, and 1 mg mL^−1^ BR, fructose content significantly increased by 14.42%, 24.78%, and 11.80%, respectively, and high fructose content was observed in 0.5 mg mL^−1^ BR (Figure 5b). Furthermore, glucose content was also significantly increased by 50.00% and 71.83% with spraying 0.1 and 0.5 mg mL^−1^ BR, respectively (Figure 5c), whereas 1 mg mL^−1^ BR reduced it by 60.54%. Similarly, spraying 0.1 and 0.5 mg mL^−1^ BR increased soluble sugar content by 7.20% and 20.59%, respectively, but decreased at 1 mg mL^−1^ BR (Figure 5d).

To elucidate this metabolic process, we analyzed the activity of sugar metabolism-related enzymes (Figure 5e–h). Compared to DS+H_2_O, spraying 0.1 and 0.5 mg mL ^1^ BR significantly suppressed SPS activity by 19% and 25%, respectively, while markedly enhancing the activity of VIN, particularly at 0.5 mg mL^−1^ BR (Figure 5g). Additionally, the SS-I and CWIN activity had no significant difference among the treatments (Figure 5f–h).

### 3.6. Transcriptome Analysis of BR-Treated Water-Deficit-Stressed Silk

**Figure 6:**
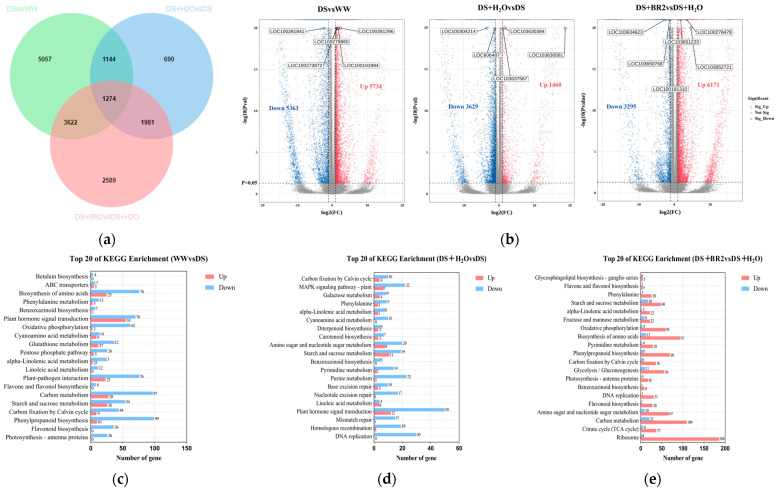
Transcriptome analysis of BR-treated, water-deficit-stressed silk. (**a**) Venn diagram showing DEG overlap among comparisons. (**b**) Volcano plot of significantly differentially expressed genes (DEGs), with | log _2_ FC | > 1 and *p*-value < 0.05. (**c**−**e**) Top 20 KEGG pathways enriched in (**c**) DS vs. WW, (**d**) DS+H_2_O vs. DS, and (**e**) DS+BR2 vs. DS+H_2_O.

To investigate the response of exogenous BR to water-deficit stress, transcriptome sequencing (RNA-seq) was performed on three treatments of WW, DS, DS+H_2_O, and DS+BR2 (0.5 mg mL^−1^), with three biological repetitions. The alignment rate of transcriptomic data to the Zm_B73 reference genome was above 85% (Table S1). Additionally, we performed qRT-PCR analysis on fifteen selected DEGs (LOC100278478, LOC100273093, LOC542091, LOC100170246, LOC103634623, LOC100272381, LOC103627433, LOC542718, LOC100383210, LOC100382364, LOC100280694, LOC100281981, LOC100191310, LOC103652721, LOC103626358) (Table S2). The results showed that these expression trends were consistent with the RNA-seq dataset, suggesting that transcriptomic data can be used for subsequent analysis (Figure S1). We identified 14555 DEGs among the DS vs. WW, DS+H_2_O vs. DS, and DS+BR2 vs. DS+H_2_O comparison groups, with 1274 DEGs common to both comparisons (Figure 6a). The volcano plot showed that the number of genes was significantly up-regulated by spraying BR, with 6171 up-regulated DEGs and 3295 down-regulated DEGs in the DS+BR2 vs. DS+H_2_O comparison (Figure 6b). KEGG (Kyoto Encyclopedia of Genes and Genomes) enrichment analysis revealed that starch and sucrose metabolism, phenylalanine, and alpha-linolenic acid metabolism were significantly enriched in the DS vs. WW, DS+H_2_O vs. DS, and DS+BR2 vs. DS+H_2_O comparisons (Figure 6c–e). Notably, downregulated DEGs dominated in DS vs. WW and DS+H_2_O vs. DS comparisons, whereas upregulated DEGs dominated in DS+BR2 vs. DS+H_2_O. Additionally, MAPK signaling pathways were enriched in the DS+H_2_O vs. DS comparison (Figure 6d), while phenylpropanoid biosynthesis and glycolysis/gluconeogenesis were enriched in the DS+BR2 vs. DS+H_2_O comparison (Figure 6e).

### 3.7. Expression of Starch and Sucrose Metabolism Genes Under BR Treatment

**Figure 7:**
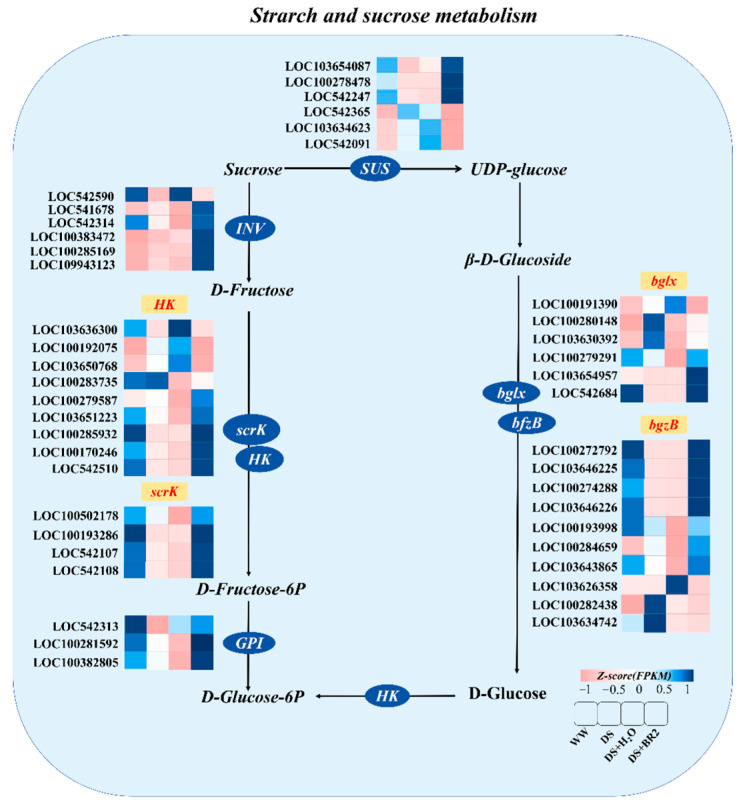
Expression of DEGs in the starch and sucrose metabolism pathway under BR treatment. The color scale indicates log _2_ (fold change).

In the starch and sucrose metabolic pathway, several DEGs were upregulated by foliar application of BR (Figure 7). BR spraying upregulated the expression of DEGs related to sucrose to glucose metabolism, involving sucrose phosphate synthase (SUS), bgzB, and bglx compared to DS and DS+H_2_O. Meanwhile, BR also significantly upregulated the key genes such as sucrose invertase (INV), hexokinase (HK), and glucose phosphate isomerase (GPI) in the process of sucrose decomposition into glucose.

### 3.8. Expression of Glycolysis/Gluconeogenesis Genes Under BR Treatment

**Figure 8:**
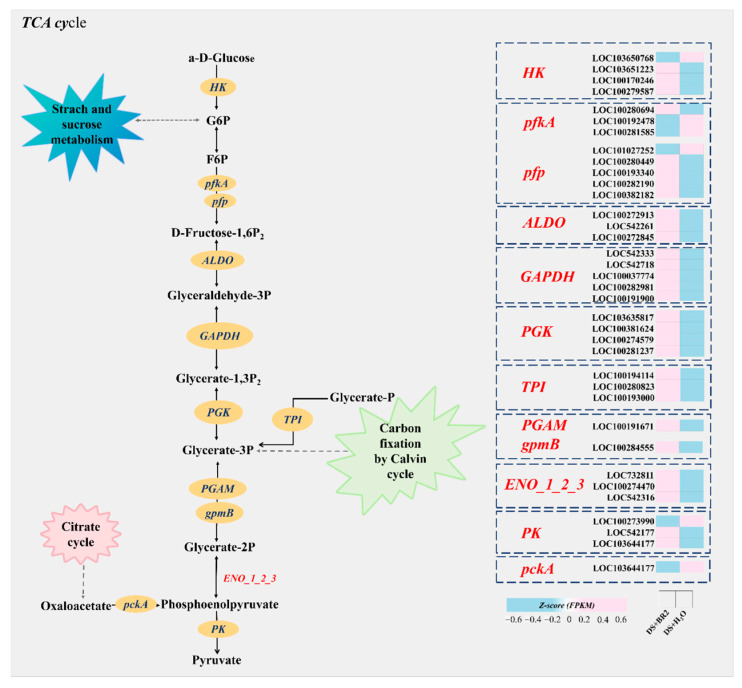
Expression of DEGs in the glycolysis/gluconeogenesis pathway under BR treatment. The color scale indicates log _2_ (fold change).

KEGG enrichment analysis of glycolysis/gluconeogenesis after BR spraying is shown in Figure 8. In the initial process of glycolysis, spraying BR significantly upregulated the expression of DEGs encoding HK. Concurrently, spraying BR upregulated the entire glycolytic pathway, including upregulating the expression of DEGs such as phosphofructokinase-1 (pfkA), pyrophosphate-dependent phosphofructokinase (pfp), aldolase (ALDO), glyceraldehyde-3-phosphate dehydrogenase (GAPDH), phosphoglycerate kinase (PGK), and triosephosphate isomerase (TPI). Thus, it promotes the activity of carbohydrate metabolism pathways related to sugar metabolism, as well as the tricarboxylic acid cycle.

## 4. Discussion

Drought stress during the pre-anthesis stage severely compromises maize reproductive success, primarily through its potent inhibition of silk elongation. This inhibition is most severe at the ear tip, where delayed silk emergence and exposure to dry conditions lead to pollination failure and prolonged ASI [11,44,45,46]. To dissect the underlying mechanisms, we imposed a controlled, moderate water deficit regime in a pot experiment. It is well known that stress of this magnitude induces adaptive metabolic changes without causing irreversible damage, thereby providing a suitable context for studying plant hormone-mediated adaptation. Within this defined stress context, the efficacy of foliar-applied BR exhibited a biphasic concentration dependence, a pattern indicative of its role as a low-dose signaling molecule in stress adaptation [47,48].

Drought triggers excessive ROS accumulation, leading to cellular damage that hinders growth processes [49,50,51,52]. In this study, BR application significantly reduced ROS accumulation (O_2−_, H_2_O_2_) while enhancing antioxidant enzyme activities (POD, SOD, and CAT), thereby mitigating membrane lipid peroxidation (Figure 2 and Figure 3). BR spraying also markedly increased proline content (Figure 4), indicating improved osmotic adjustment and cellular hydration under water deficit conditions. Our transcriptomic analysis provides a mechanistic link to these physiological observations, revealing a significant enrichment of the phenylpropanoid biosynthesis pathway (e.g., PAL, C4H, CCR; Figure S2). The upregulation of this pathway suggests BR may enhance ROS scavenging capacity and potentially reinforce cell walls not only via enzymatic activity but also through potential stimulation of non-enzymatic defense mechanisms [53,54,55]. This coordinated enhancement of both enzymatic and non-enzymatic antioxidant systems establishes a robust defensive front, creating a more favorable cellular environment for silk elongation.

Importantly, unlike prior research focused on improving drought tolerance via photosynthesis or osmotic adjustment, our study further found that BR may alleviate the constraints of energy supply and redox balance by reprogramming silk sucrose metabolism [56,57]. Sucrose metabolism is not only pivotal for carbon and energy supply but also serves as a key regulator of cellular redox homeostasis under stress [8,58,59]. In our study, water deficit-induced sucrose accumulation (Figure 5a) may be attributed to increased SPS activity and suppressed VIN activity (Figure 5e,g). This created a metabolic bottleneck, restricting hexose flux. The resulting limitation may constrain both glycolytic ATP supply and substrate availability for the pentose phosphate pathway (PPP), a crucial source of NADPH for antioxidant regeneration [60,61,62]. Consequently, this dual deficiency in energy and reducing capacity exacerbates ROS accumulation and oxidative damage, further inhibiting silk elongation.

However, the application of BR effectively alleviated this metabolic restriction. It significantly inhibited SPS activity while enhancing VIN activity, thereby redirecting carbon flux toward hexose (e.g., glucose and fructose) production (Figure 5). The increase in hexose not only contributes to osmotic regulation and cell expansion but may also promote glycolysis, producing ATP for biosynthesis [63,64,65,66]. In addition, the increase in hexose may also provide substrates for PPP, promoting the production of NADPH [67]. NADPH is crucial for the regeneration of antioxidant systems, such as the ascorbic acid glutathione cycle [68,69]. Therefore, BR may have established a virtuous cycle by restarting carbon flow, restoring redox homeostasis, and supporting cell elongation. Integration of transcriptomic and physiological data further supports this result and reveals BR’s function. Although genes involved in sucrose metabolism were broadly upregulated (INV, HK, GPI, GAPDH, PGK, TPI; Figure 7 and Figure 8), BR selectively enhanced VIN activity without increasing CWIN activity (Figure 5g–h), which is consistent with the upregulation of the VIN encoding gene in transcriptome data. This indicates that BR-mediated relief prioritizes the mobilization of intracellular (vacuolar) sucrose reserves for metabolic utilization rather than modulating apoplastic sucrose unloading. Notably, this regulation exhibits gene family member specificity. This indicates that the BR signal does not simply activate the entire pathway. Within sucrose-metabolizing gene families, different paralogs (e.g., in INV, HK, and SUS families) showed varying degrees of responsiveness to BR (Figure 7 and Figure 8). For example, the upregulation of SUS paralogs suggests a synergistic shift toward the cleavage direction, yielding UDP-glucose, which is a key precursor for cell wall biosynthesis [70,71,72]. Together with the concurrently enhanced phenylpropanoid pathway, this indicates that BR-mediated reprogramming not only resolves the energy crisis but also coordinates the supply of building materials to reinforce silk structure under stress, offering defined candidate genes for future validation.

## 5. Conclusions

Silk elongation suppression is a major cause of maize grain yield loss under reproductive water-deficit stress. Foliar-applied brassinolide (BR) effectively mitigated water deficit-induced adverse effects, with 0.5 mg mL^−1^ BR being the most effective. Physiological analyses revealed that the mitigation of water deficit-inhibited silk elongation by foliar-applied BR was attributed to orchestrated physiological improvements through increased antioxidant enzyme activities, including catalase (CAT), superoxide dismutase (SOD), and peroxidase (POD), and boosted proline accumulation. Importantly, the application of BR reprogrammed sugar metabolism, shifting the balance from sucrose to hexose accumulation in silk. Transcriptome data further supported these findings, with BR-induced upregulation of the expression of genes in sucrose metabolism and glycolysis pathways. The concomitant upregulation of phenylpropanoid biosynthesis genes was associated with the mitigation of water-deficit stress by BR application, potentially mediated by secondary metabolites. Overall, foliar-applied BR alleviated water deficit-induced silk elongation inhibition mainly by enhancing antioxidant capacity, maintaining osmotic adjustment, and reprogramming sucrose metabolism toward hexose accumulation to support energy flow in reproductive tissues, likely involving phenylpropanoid secondary defense. Beyond its foliar application, this study provides mechanistic insights and molecular targets for developing water deficit-resilient maize cultivars.

## Data Availability

The original contributions presented in this study are included in the article. Further inquiries can be directed to the corresponding author.

## Supplementary Materials

The following supporting information can be downloaded at: https://www.mdpi.com/article/10.3390/plants15010139/s1, Figure S1: Validation of RNA-seq results by qRT-PCR; Figure S2: The expression of DEGs related to phenylpropanoid biosynthesis metabolic pathways after BR spraying; Table S1: Comparison ratio of transcriptome data with reference genome; Table S2: The list of primers for qRT-PCR validation of selected genes.
