# Supplementary material for: Brassinolide Alleviates Maize Silk Growth Under Water Deficit by Reprogramming Sugar Metabolism and Enhancing Antioxidant Defense
Source: Plants (Basel). 2026 Jan 3;15(1):139. doi: 10.3390/plants15010139 (PMC12787882; doi:10.3390/plants15010139)

Table. S1 Comparison ratio of transcriptome data with reference genome

Sample name	Valid Reads	mapped N/A	Unique mapping reads	Unique mapping%
WW_1	34372166	87.12%	28899400	84.08%
WW_2	36875958	87.09%	31088822	84.31%
WW_3	42910518	86.85%	36144366	84.23%
DS_1	35717044	86.18%	29204276	81.77%
DS_2	43542730	85.97%	35621678	81.81%
DS_3	37030698	86.27%	30549913	82.50%
DS+H_2O_1	34494922	85.85%	28132821	81.56%
DS+H_2O_2	36117780	85.30%	29409895	81.43%
DS+H_2O_3	35231562	85.50%	28739567	81.57%
DS+BR2_1	49567392	88.52%	42073458	84.88%
DS+BR2_2	37216466	87.46%	31388182	84.34%
DS+BR2_3	36600306	87.69%	30887750	84.39%

Notes: WW, well-watered; DS, drought stressed; DS+H_2O, as spraying control, spraying distilled water under drought stress; DS+BR2, spraying 30 mM BR under drought stress

Table. S2 The list of primers for qRT-PCR validation of selected genes.

Gene id	Forward primer	Reverse primer
Actin	TGACTGGTGTTGTCCGAATAGT	AATGCTGGGGAAGACAGCTC
LOC100278478	ACCTCTCAACACCCCATCTG	CCGCTAACAAATACCTCCGC
LOC100273093	CTGTGTCAAGCCGCTAACTC	AAGGGCGCCGAAAATACAAG
LOC542091	TCAAAGCTCCCTGCTGACAC	CTGGCGCCTGAATGATGTCT
LOC100170246	TGACTCTTCGCATGACCTCA	TCCAGGGAAGTATCTGCGAC
LOC103634623	GCGTGCCTTGTATAAAGTCGG	CAGTGGTCAGTGGGTCGTTT
LOC100272381	AAGTCAAGTCAAGCACCGGA	ACTACTGCAGGACGTACGGA
LOC103627433	TCTTGGAGGGCAGCTCTTAC	GAAGTTGCCACCGTGAAGAG
LOC542718	TCACTGACAAGGACAAGGCT	CTGAGGCTACTTCCAACCCA
LOC100383210	TGAACCCTAGTAACGCGTCG	GCCATGGCACCAGCTAGTAT
LOC100382364	GCGGCGTACTGTACTCACTA	CGAGTGCCTAACGTTACCGAG

LOC100280694	GGCCACAAGGTTTTATGCCG	CACGGATACGGCTCTAGCAA
LOC100281981	GTGCTGTGTTTCATCCAGTGT	ATGGGCGATTACAAAAGCACG
LOC100191310	GCGGATTCAATGGCGAGAAC	TTCAGTAGTCAGCACGCAGG
LOC103652721	CTCACCGTCTGCGTTGATGT	ACCTACGATAACAGGGCAGC
LOC103626358	TAGGACACGGACGAGGGTT	ATGCATACGTACCTCGTGAC

Figure. S1. Validation of RNA-seq results by qRT-PCR.

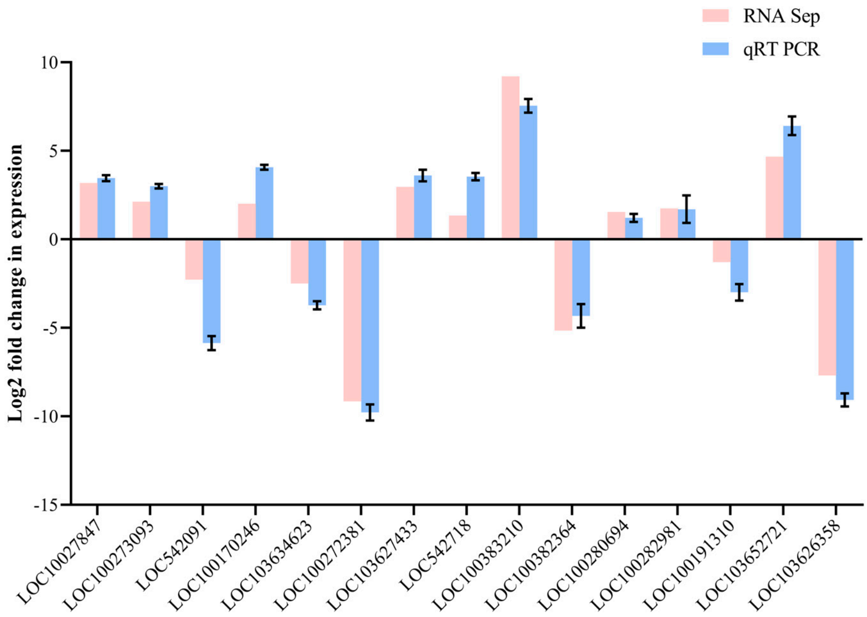

Notes: Gene expression trends for fifteen selected differentially expressed genes (DEGs) identified from the DS+BR2 vs DS+H_2O comparison were validated by qRT-PCR. Data are means ± SD (n=3).

Figugar S2. The expression of DEGs related to phenylpropanoid biosynthesis metabolic pathways after BR spraying.

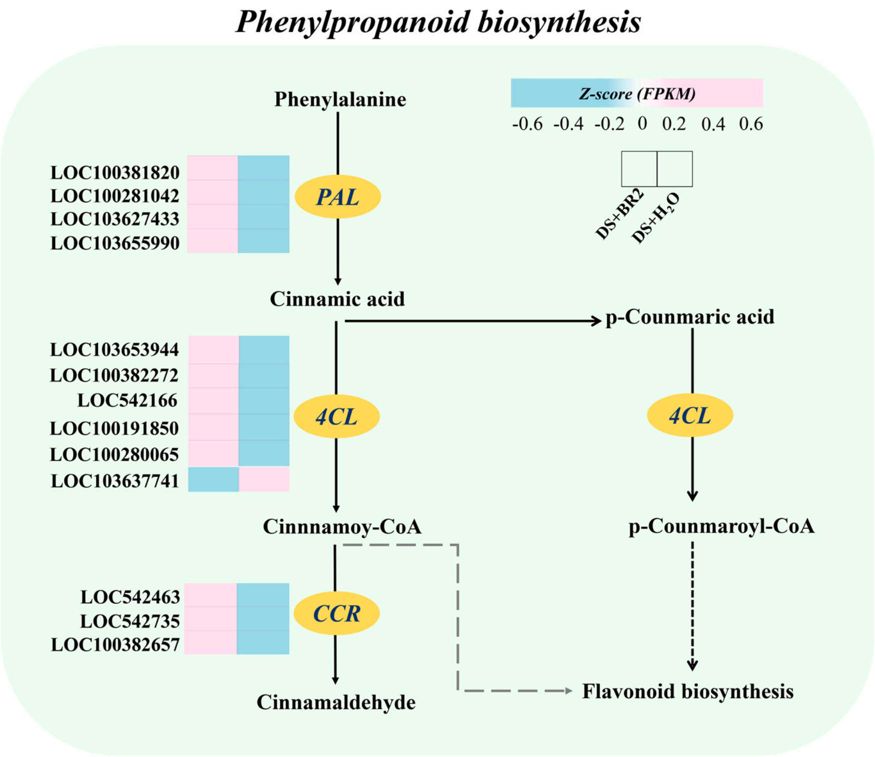

Supplement: Supplementary file 1 [file plants-15-00139-s001.zip › plants-4009283-supplementary.pdf]
